# Antibiotic Susceptibility Profiles and Determinants of Septic Abortion Among Women Admitted for Abortion Care in Ugandan Satellite Teaching Hospitals: A Two‐Center Cross‐Sectional Study

**DOI:** 10.1002/hsr2.72062

**Published:** 2026-03-11

**Authors:** Guled Bashir Gaboose, Marie Pascaline Sabine Ishimwe, Richard Mulumba, Ahmed Kiswezi Kazigo, Theodore Nteziyaremye, Theoneste Hakizimana

**Affiliations:** ^1^ Department of Obstetrics and Gynecology Kampala International University Ishaka Uganda; ^2^ Department of Pediatrics and Child Health Kampala International University Ishaka Uganda; ^3^ Department of Surgery Kampala International University Ishaka Uganda; ^4^ Department of Sciences University of Rwanda Kigali Rwanda

**Keywords:** antibiotic susceptibility profile, bacterial isolates, determinants, prevalence, Septic abortion

## Abstract

**Background and Aims:**

Septic abortion remains a major cause of maternal morbidity and mortality in low‐ and middle‐income countries. This study aimed to determine the prevalence, antibiotic susceptibility profiles, and determinants of septic abortion among women admitted for abortion care in Ugandan satellite teaching hospitals.

**Methods:**

This two‐center cross‐sectional study was conducted at Fort Portal and Hoima Regional Referral Hospitals between May and September 2023. Consecutive women admitted for abortion care were enrolled. Participants were initially identified based on clinical signs and symptoms suggestive of septic abortion, including fever ( ≥ 38°C) and features of genital tract infection. Microbiological diagnosis was subsequently confirmed using culture and antibiotic susceptibility testing. Endocervical swabs from clinically suspected cases were cultured, and antimicrobial susceptibility testing was performed using the Kirby–Bauer disk diffusion method and interpreted according to Clinical and Laboratory Standards Institute (CLSI) guidelines. Logistic regression was used to identify factors associated with septic abortion.

**Results:**

The prevalence of septic abortion was 13.0% (27/207). The most frequently isolated organisms were *Escherichia coli* and *Staphylococcus aureus*. High resistance was observed to commonly used antibiotics, whereas higher susceptibility was noted for reserve antibiotics. Rural residence and lack of contraceptive use were independently associated with septic abortion (adjusted odds ratios reported with 95% confidence intervals).

**Conclusions and Recommendations:**

Septic abortion remains common among women admitted for abortion care in these Ugandan hospitals and is characterized by substantial antimicrobial resistance to commonly used agents. Strengthening access to effective contraception, promoting early care‐seeking, and guiding empiric treatment using local antimicrobial susceptibility patterns are essential to reduce morbidity.

**Abbreviations**
AMR: Antimicrobial resistance
ETB: Institutional Obstetrics and Gynaecology
FRRH: Fort Portal Regional Referral Hospital
HRRH: Hoima Regional Referral Hospital
IREC KIU: Institutional Research Ethics Committee Kampala International
PAC: Post abortion care
SSA HMIS: Sub‐Saharan Africa Health management information system

## 1 Introduction

Septic abortion is a pregnancy‐related infection of the uterus and its contents occurring during or shortly after spontaneous or induced abortion, typically characterized by fever, lower abdominal/pelvic pain, uterine tenderness, and/or foul‐smelling vaginal discharge [1, 2, 3]. Globally, 46 million abortions occur annually, with 19 million being unsafe [4]. Unsafe abortions contribute significantly to maternal morbidity and mortality, accounting for 20–30% of reproductive tract infections and 5% of chronic infections in women of reproductive age [5]. Post‐abortion infections cause 22.1% of severe maternal illnesses worldwide and 9.5% of the global maternal infection burden [6].

The prevalence of septic abortion varies across regions. In Iran, it was reported at 22.5% among women admitted with infectious abortion complications [7]. In sub‐Saharan Africa, where 75.6% of abortions are considered unsafe, the incidence of abortion varies, with rates as low as 2.4% reported in Tanzania [8, 9]. In Uganda, infection‐related abortions constitute 16% of severe abortion morbidities [10].

Septic abortion is commonly caused by polymicrobial infections, including infections caused by *E. coli*, *Clostridium perfringens*, *S. aureus*, and *Pseudomonas aeruginosa* [11, 12]. These bacteria originate from the commensal vaginal flora and sexually transmitted pathogens, leading to severe complications such as septic shock and maternal death [13, 14].

In Nigeria, a hospital‐based study of women with septic incomplete abortion in Port Harcourt reported *E. coli* as the most common isolate (49.2%), followed by *S. aureus* (37.1%), while anaerobes such as *Bacteroides* (3.8%) and *Clostridium* species (2.3%) were identified less frequently [15]. In a study by Patience et al., *Morganella* spp. were found to be 100% susceptible to ciprofloxacin and 100% resistant to cefotaxime, gentamycin, and ampicillin [16].

Despite increasing antibiotic resistance, empirical therapy typically includes combinations such as gentamicin and clindamycin or broad‐spectrum monotherapy with imipenem or piperacillin–tazobactam [17, 18]. However, surgical evacuation of necrotic tissue remains the cornerstone of treatment [5].

Several factors are associated with septic abortion. Studies in India and Ethiopia report increased risk among younger women, rural dwellers, multiparous women, and those in the first trimester [19, 20]. In Uganda, delays in seeking care and limited access to microbial culture testing further complicate management, as septic abortion remains a leading cause of maternal mortality [21].

In this study, the prevalence of septic abortion refers to the proportion of women admitted for abortion care during the study period who met the clinical definition of septic abortion. Given the high burden of septic abortion, this study aims to determine its prevalence, bacterial susceptibility patterns, and determinants of septic abortion among women admitted to satellite teaching hospitals in Uganda. The findings will guide evidence‐based interventions to improve diagnosis, treatment, and maternal outcomes.

## 2 Materials and Methods

### 2.1 Study Design and Setting

This descriptive two center cross‐sectional study was conducted at the gynecological clinics and wards of Fort Portal and Hoima Regional Referral Hospitals from May, 2023 to September, 2023.

Fort Portal Regional Referral Hospital (FRRH) is located within Fort Portal Municipality, approximately 295 kilometers west of Kampala, Uganda′s capital city. It has a bed capacity of 384 and serves the entire Tooro region, which includes eight districts (Bundibugyo, Kabarole, Kyenjojo, Kasese, Kamwenge, Kyegegwa, Bunyangabu, and Ntoroko) as well as parts of the Eastern Democratic Republic of Congo (DRC).

Hoima Regional Referral Hospital (HRRH) is a recognized institution with a capacity of 300 beds. It provides a variety of medical services, including obstetrics and gynecology, general surgery, internal medicine, and pediatrics. It is located in Hoima City, Western Uganda, approximately 230 kilometers northwest of Kampala. It is bordered by Kyankwanzi District to the east, Kibaale District to the south, Buliisa District to the north, Masindi District to the northeast, Ntoroko District to the southwest, and the Democratic Republic of Congo across Lake Albert to the west.

Both Fort Portal and Hoima Regional Referral Hospitals offer general and specialized medical services and serve as teaching sites for Kampala International University and other institutions. Their laboratories are equipped to conduct various tests, including culture and sensitivity testing.

### 2.2 Inclusion Criteria

All women who were diagnosed with abortion attending the gynecological clinics and wards of FRRH and HRRH and who consented to participate in this study were included.

### 2.3 Exclusion Criteria

Women who were already receiving antibiotic treatment or were unconscious were excluded from the study.

### 2.4 Sample Size Determination

This was a cross‐sectional study. A priori sample size estimation was performed to ensure adequate precision for estimating the prevalence of septic abortion and sufficient power to explore associated factors. The sample size for the study was determined using Kish–Leslie's (1965) formula for a single proportion based on the prevalence of septic abortion.

$$n=\frac{z^{2}p\left(1-p\right)}{d^{2}}$$

where,

*n* = Minimum sample size

*Z* = the table value for the standard normal deviation corresponding to 95% significance ( = 1.96)

*P* = Prevalence of septic abortion of 16% in Uganda [10]

*d* = Margin error, set at 0.05

$$n=\frac{z^{2}p\left(1-p\right)}{d^{2}}$$

$$n=\frac{{\left(1.96\right)}^{2}*0.16\left(1-0.16\right)}{{\left(0.05\right)}^{2}}$$

$$n=\frac{3.8416x0.16x0.84}{0.0025}$$

n=207

Therefore, *n* = 207 participants.

The sample size required for this study was 207 women with abortions at two tertiary hospitals.

### 2.5 Sampling Technique

A consecutive sampling method was employed until the required sample size of 207 was attained. The sample size was deemed sufficient, and all participants met the eligibility criteria.

### 2.6 Diagnosis of Septic Abortion

Clinical assessment was performed by trained clinicians/research staff using a standardized tool. Septic abortion was defined clinically as abortion complicated by signs of genital tract infection, including fever ( ≥ 38°C), chills, lower abdominal/pelvic pain, uterine tenderness, and/or foul‐smelling vaginal or cervical discharge, with or without systemic features of infection. Endocervical swabs were collected only from participants meeting the clinical definition of septic abortion for culture and susceptibility testing as part of routine evaluation of suspected infection [3].

### 2.7 Study Procedure and Specimen Collection

All women diagnosed with abortion at the gynecological clinics and wards of Fort Portal Regional Referral Hospital (FRRH) and Hoima Regional Referral Hospital (HRRH) were informed about the study by the principal investigator and trained research assistants. Written informed consent was obtained, and participants completed a pretested questionnaire. A routine medical history and physical examination were conducted, including assessment for fever or chills, lower abdominal pain, uterine tenderness, vaginal bleeding, foul‐smelling vaginal discharge, and findings on speculum examination.

#### 2.7.1 Specimen Collection

Women presenting with clinical signs and symptoms suggestive of septic abortion were further informed about the specimen collection procedure. Under aseptic conditions, a sterile Cusco′s speculum was inserted to visualize the cervix. The cervix was first cleaned using a sterile swab to remove vaginal secretions, which was discarded. A second sterile swab was then inserted into the cervical canal and rotated for 10–30 s to obtain an adequate endocervical specimen. Specimen collection was performed before uterine evacuation for retained products of conception.

#### 2.7.2 Specimen Transport

Immediately after collection, the swab was placed into a sterile Bijou bottle containing freshly prepared Stuart′s transport medium. Air was expelled, the swab stick was broken at the midpoint, and the container was tightly sealed. Specimens were placed in sealed plastic bags and transported to the microbiology laboratory at temperatures ranging from 2°C to 30°C.

#### 2.7.3 Culture and Incubation

In the laboratory, clinical specimens were inoculated onto blood agar, MacConkey agar, and chocolate agar (OXOID Ltd., UK) using standard aseptic techniques. Inoculation was performed with a sterile wire loop that was flamed to red‐hot over a Bunsen burner and allowed to cool before streaking the plates in a zigzag pattern to obtain discrete colonies. Culture plates were incubated at 37°C for 18–24 h; chocolate agar plates were incubated in a candle jar to provide an approximate 10% carbon dioxide (CO₂) atmosphere.

#### 2.7.4 Identification of Bacterial Isolates

After incubation, plates were examined macroscopically for colony morphology, including size, shape, texture, and hemolytic patterns. Alpha‐hemolysis was identified by green discoloration on blood agar, while beta‐hemolysis was identified by clear zones of lysis. For microscopic examination, bacterial smears were prepared on grease‐free slides using normal saline, air‐dried, heat‐fixed, and Gram‐stained following standard procedures (Cheesbrough, 2006). The staining process involved sequential application of crystal violet, Lugol′s iodine, 50% acetone–alcohol, and neutral red, each for approximately 60 s with water rinses between steps. Gram reaction and cellular morphology (e.g., cocci or rods arranged in pairs, chains, or clusters) were assessed under light microscopy.

#### 2.7.5 Interpretation of Culture Results

Culture findings were interpreted as significant growth when pathogenic organisms were isolated and classified based on Gram reaction and morphology (e.g., Gram‐negative rods or Gram‐positive cocci). No significant growth was defined as the absence of bacterial growth after 48 h of incubation.

#### 2.7.6 Antibiotic Susceptibility Testing

Antibiotic susceptibility testing was performed on confirmed bacterial isolates using the modified Kirby–Bauer disk diffusion method on Mueller–Hinton agar [22]. The following antibiotic disks were tested: ampicillin, chloramphenicol, imipenem, piperacillin/tazobactam, ciprofloxacin, co‐trimoxazole, cefuroxime, erythromycin, azithromycin, vancomycin, doxycycline, and gentamicin. After placement of antibiotic disks on the inoculated media, plates were incubated at 37°C for 18–24 h. Zones of inhibition were measured in millimeters using a ruler and interpreted as susceptible, intermediate, or resistant according to standard clinical microbiology guidelines.

### 2.8 Study Variables

Independent variables included sociodemographic, obstetric and gynecological factors associated with septic abortion. The dependent variable was a septic abortion as a binary variable.

### 2.9 Quality Control

The inclusion and exclusion criteria were strictly adhered to ensure the collection of valid data. All completed questionnaires were carefully checked to confirm that no essential information was missing. Research assistants received thorough training before data collection to ensure the proper use of data collection tools and adherence to ethical standards.

After collection, the endocervical swabs were placed into Bijou bottles containing freshly prepared Stuart′s transport medium, ensuring that air was expelled from the bottles. The bottles were then securely sealed with their covers and maintained at temperatures ranging from 2°C to 30°C until they were tested. The samples were labeled using a numerical code corresponding to each participant, allowing easy identification. To ensure quality control, 10% of the samples were sent to the main laboratory at Kampala International University Teaching Hospital. For participants who were not familiar with the local language or English, translated versions of the questionnaire were provided to ensure understanding and accurate responses.

### 2.10 Data Analysis

Participants were recruited based on the presence of clinical signs and symptoms suggestive of septic abortion, including fever, lower abdominal or pelvic pain, uterine tenderness, and foul‐smelling vaginal discharge. Microbiological confirmation was subsequently performed, and bacteriological diagnosis was established through culture and antibiotic susceptibility testing of endocervical swabs.

Data were entered in Microsoft Excel (2016) and exported to IBM SPSS Statistics (version 22.0; IBM Corp., Armonk, NY, USA) for analysis. Continuous variables were summarized using means (standard deviations) for approximately normally distributed data and medians (interquartile ranges) for skewed data. Categorical variables were summarized using frequencies and percentages and reported with numerators and denominators. Descriptive analyses were used to estimate the prevalence of septic abortion, describe bacterial isolates identified through culture, and summarize antibiotic susceptibility profiles. The prevalence of septic abortion was calculated as the proportion of participants with culture‐confirmed infection among the total number of study participants. Bacterial isolates were summarized as frequencies and percentages among women with culture‐confirmed septic abortion, and susceptibility results were categorized as susceptible, intermediate, or resistant according to CLSI standards [22].

Factors associated with septic abortion were assessed using Univariable and multivariable binary logistic regression. Variables with *p* ≤ 0.20 at Univariable analysis and/or strong biological plausibility were included in the multivariable model. Results are presented as crude and adjusted odds ratios with 95% confidence intervals. All tests were two‐sided, and *p* ≤ 0.05 was considered statistically significant.

## 3 Results

### 3.1 Basic Characteristics of Participants

A total of 358 women admitted for abortion care were approached for participation. Of these, 12 declined to provide consent. Among the 346 patients who consented, 139 were excluded (134 due to recent antibiotic use within the preceding 48 h, and 5 who were unable to complete the interview). The final sample comprised 207 participants. The majority of the respondents were aged 20–29 years (109, 52.7%), resided in rural areas (122, 58.9%), were married (160, 77.3%), and had attained a primary level of education (102, 49.3%). Most were engaged in subsistence farming (93, 44.9%) and reported a monthly income below 250,000 Ugandan Shillings (168, 81.2%). A large proportion identified as Christian (189, 91.3%), lived in extended family households (136, 65.7%), and resided in homes with fewer than five household members (116, 56%). (Table 1). Baseline characteristics are presented in Table 1.

**Table 1:** Sociodemographic characteristics of the study participants (*N* = 207).

Variables	Categories	Frequency (*n*)	Percentage (%)
Age	< 20	27	13.0
	**20–29**	**109**	**52.7**
	30–39	59	28.5
	≥ 40	12	5.8
Residence	**Rural**	**122**	**58.9**
	urban	85	41.1
Marital status	Single	47	22.7
	**Married**	**160**	**77.3**
Education level	None	12	5.8
	**Primary**	**102**	**49.3**
	Secondary	71	34.3
	Tertiary	22	10.6
Occupation	Housewife	32	15.5
	**Peasant**	**93**	**44.9**
	Business	49	23.7
	Students	14	6.8
	Professional	19	9.2
Income	None	15	7.2
	**< 250,000**	**168**	**81.2**
	≥ 250,000	24	11.6
Religion	Muslim	18	8.7
	**Christian**	**189**	**91.3**
Family type	Nuclear family	71	34.3
	**Extended Family**	**136**	**65.7**
House size	**< 5members**	**116**	**56**
	≥ 5members	91	44

Abbreviation: UGX = Ugandan shillings.

### 3.2 Prevalence of Septic Abortion Among Women Admitted for Abortion Care in Ugandan Satellite Teaching Hospitals (*N* = 207)

**Figure 1:**
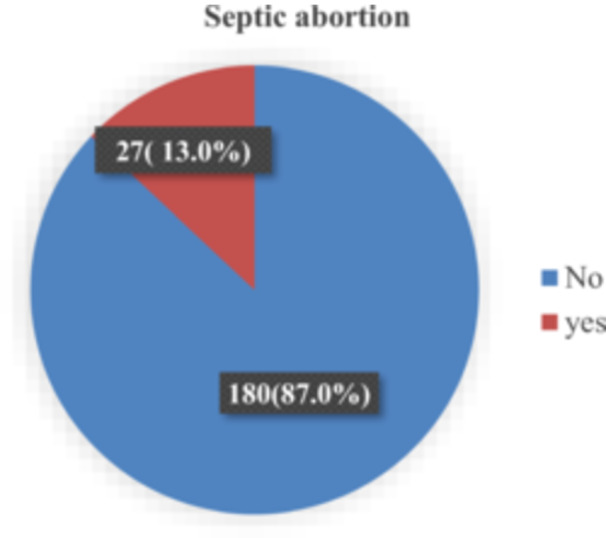
Pie chart representing the prevalence of septic abortion among mothers with abortion attending 2 satellite teaching hospitals in Uganda.

Of the 207 patients with abortion included in the study at the two satellite teaching hospitals, 27 (13.0%) were diagnosed with septic abortion, while 180 (87.0%) did not have septic abortion. (Figure 1).

### 3.3 Bacterial Isolates from Women with Septic Abortion Attending Fort Portal and Hoima Regional Referral Hospitals (*N* = 27)

**Figure 2:**
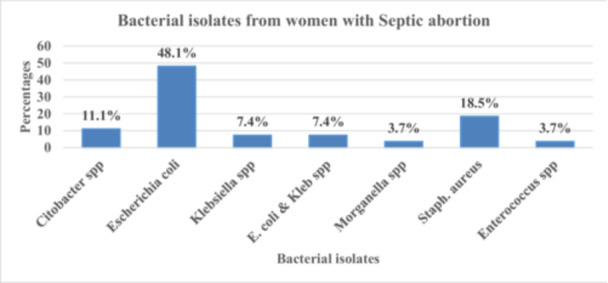
Bacterial isolates from women with septic abortion (n = 27) at Fort Portal and Hoima Regional Referral Hospitals. Bars represent the number (and percentage) of isolates identified by culture from endocervical swabs.

The bacterial isolate for the majority of samples among mothers with septic abortion was *E. coli* 13 (48.1%), followed by *S*. *aureus* 5 (18.5%), *Citrobacter* spp. 3 (11.1%), *Klebsiella* spp. 2 (7.4%), *E*. *coli* & *Klebsiella* 2 (7.4%), *Enterococcus* spp. 1 (3.7%), and *Morganella* spp. 1(3.7%). (Figure 2).

### 3.4 Antibiotic Susceptibility Profiles of Septic Abortion Among Women Admitted for Abortion Care at Ugandan Satellite Teaching Hospitals

Most of the E. coli strains isolated were sensitive to chloramphenicol 12(92.3%), imipenem 13(100.0%), piperacillin/Tazobactam 13(100.0) and vancomycin 12(92.3) (Table 2).

**Table 2:** Antibiotic susceptibility profiles of bacterial isolates from women with septic abortion (n = 27).

Organisms isolated		Antibiotics, *n* = 27[%]									
		Cipro	Chlora	Imipen	Genta	Ampi	Cefotax	Augme	Azithro	Peperac	Vanco
*Citobacter* spp	S	0 (0.0)	1 (33.3)	2 (66.7)	2 (66.7)	0 (0.0)	0 (0.0)	0 (0.0)	0 (0.0)	2 (66.7)	2 (66.7)
	I	1 (33.3)	0 (0.0)	0 (0.0)	0 (0.0)	0 (0.0)	0 (0.0)	1 (33.3)	0 (0.0)	0 (0.0)	0 (0.0)
	R	2(66.7)	2 (66.7)	1 (33.3)	1 (33.3)	3 (100.0)	3 (100.0)	2 (66.7)	3 (100.0)	1 (33.3)	1 (33.3)
*E. coli*	S	7 (50.0)	14 (93.3)	15 (100.0)	8 (57.1)	4 (28.6)	6 (42.9)	5 (35.7)	6 (42.9)	15 (100.0)	13 (92.9)
	I	1 (7.1)	0 (0.0)	0 (0.0)	0 (0.0)	0 (0.0)	2 (14.3)	1 (7.1)	0 (0.0)	0 (0.0)	0 (0.0)
	R	6 (42.9)	1 (6.7)	0 (0.0)	6 (42.9)	10 (71.4)	6 (42.9)	8 (57.1)	7 (57.1)	0 (0.0)	1 (7.1)
*Morganella* spp.	S	1 (100.0)	1 (100.0)	1 (100.0)	0 (0.0)	0 (0.0)	0 (0.0)	1 (100.0)	0 (0.0)	1 (100.0)	1 (50.0)
	I	0 (0.0)	0 (0.0)	0 (0.0)	0 (0.0)	0 (0.0)	0 (0.0)	0 (0.0)	0 (0.0)	0 (0.0)	0 (0.0)
	R	0 (0.0)	0 (0.0)	0 (0.0)	1 (50.0)	1 (100.0)	1 (100.0)	0 (0.0)	1 (100.0)	0 (0.0)	0 (0.0)
*S. aureus*	S	2 (40.0)	4 (80.0)	5 (100.0)	3 (60.0)	1 (20.0)	2 (40.0)	2 (40.0)	4 (80.0)	5 (100.0)	5 (100.0)
	I	2 (40.0)	0 (0.0)	0 (0.0)	0 (0.0)	0 (0.0)	0 (0.0)	0 (0.00	0 (0.0)	0 (0.0)	0 (0.0)
	R	1 (20.0)	1 (20.0)	0 (0.0)	2 (40.0)	4 (80.0)	3 (60.0)	3 (60.0)	1 (20.0)	0 (0.0)	0 (0.0)
*Enterococcus* spp	S	1 (100.0)	1 (100.0)	1 (100.0)	0 (0.0)	1 (100.0)	0 (0.0)	1 (100.0)	0 (0.0)	1 (100.0)	0 (0.0)
	I	0 (0.0)	0 (0.0)	0 (0.0)	0 (0.0)	0 (0.0)	0 (0.0)	0 (0.0)	0 (0.0)	0 (0.0)	0 (0.0)
	R	0 (0.0)	0 (0.0)	0 (0.0)	1 (100.0)	0 (0.0)	1 (100.0)	0 (0.0)	1 (100.0)	0 (0.0)	1 (100.0)
*Klebsiella* spp	S	2 (66.7)	2 (66.7)	3 (100.0)	1 (33.3)	0 (0.0)	0 (0.0)	1 (33.3)	0 (0.0)	2 (66.7)	3 (100.0)
	I	0 (0.0)	0 (0.0)	0 (0.0)	0 (0.0)	0 (0.0)	0 (0.0)	0 (0.0)	0 (0.0)	0 (0.0)	0 (0.0)
	R	1 (33.3)	1 (33.3)	0 (0.0)	2 (66.7)	3 (100.0)	3 (100.0)	2 (66.7)	3 (100.0)	1 (33.3)	0 (0.0)

Abbreviations: Ampi, Ampicillin; Augme, Augmentin; Azithro, Azithromycin; Cefotax, Cefotaxime; Chlora, Chloramphenicol; Cipro, Ciprofloxacin; Genta, Gentamycin; Imipen, Imipenem; Peperac, Peperacillin Tazobactam; Vanco, Vancomycin; S = sensitive, I = intermediate Sensitive, R = resistant.

### 3.5 Determinants of Septic Abortion Among Women Admitted for Abortion Care in Ugandan Satellite Teaching Hospitals (*N* = 207)

Determinants of septic abortion were assessed by comparing women with septic abortion to women admitted for abortion care without septic abortion.

The results from the bivariate analysis revealed that rural area, occupation, lack of monthly income, gestational age less than 14 weeks, induced abortion, evacuation of the uterus from home, evacuation of the uterus from a health center and lack of contraceptive use were associated with septic abortion among women admitted for abortion care at two satellite teaching hospitals in Uganda (Table 3). After removing confounders from the multivariate analysis at 95% CI with a *p* ≤ 0.05 as a statistically significant level, rural residence and lack of contraceptive use were independently associated with septic abortion. Specifically, women from rural areas (aOR=3.775, 95% CI: 1.056–13.488, *p* = 0.041) and those with a lack of contraceptives use (aOR=6.986, 95% CI: 1.933–25.25, *p* = 0.003) were 4 and 7 times more likely to have a septic abortion, respectively (Table 4).

**Table 3:** Bivariate analysis of factors associated with septic abortion among women admitted for abortion care in Ugandan satellite teaching hospitals (N = 207).

Variables	Categories	Septic abortion		cOR [95%CI]	*p*‐value
		No (*n* = 180)	Yes (*n* = 27)		
Age	< 20	20 (74.1)	7 (25.9)	1.75 (0.306–10.022)	0.53
	20–29	96 (88.1)	13 (11.9)	0.677 (0.133–3.438)	0.638
	30–39	54 (91.5)	5 (8.5)	0.4639 (0.079–2.727)	0.395
	≥ 40	10 (83.3)	2 (16.7)	Ref	
Residence	**Rural**	**100 (82.0)**	**22 (18.0)**	**3.52 (1.276–9.709)**	**0.015** *
	urban	80 (94.1)	5 (5.9)	Ref	
Marital status	Single	39 (81.8)	8 (18.2)	1.649 (0.668–2.47)	0.278
	Married	141 (88.1)	19 (11.9)	Ref	
Education level	None	10 (83.3)	2 (16.7)	4.2 (0.339–51.981)	0.264
	Primary	86 (84.3)	16 (15.7)	3.907 (0.49–31.144)	0.198
	Secondary	63 (88.7)	8 (11.3)	2.667 (0.315–22.591)	0.368
	Tertiary	21 (95.5)	1 (4.5)	Ref	
Occupation	Housewife	29 (90.6)	3 (9.4)	0.552 (0.1–3.059)	0.496
	Peasant	79 (84.9)	14 (15.1)	0.945 (0.243–3.674)	0.935
	**Business**	**48 (98.0)**	**1 (2.0)**	**0.111 (0.011–1.145)**	**0.065**
	**Students**	**8 (57.1)**	**6 (42.9)**	**4 (0.788–20.316)**	**0.095**
	Professional	16 (84.2)	3 (15.8)	Ref	
Income	**None**	**9 (60.0)**	**6 (40.0)**	**15.333 (1.611–145.901)**	**0.018**
	< 250,000 Ugx	148 (88.1)	20 (11.9)	3.108 (0.398–24.285)	0.280
	≥ 250,000 Ugx	23 (95.8)	1 (4.2)	Ref	
Religion	Muslim	17 (94.4)	1 (5.6)	0.369 (0.047–2.89)	0.342
	Christian	163 (86.2)	26 (13.8)	Ref	
Family type	Nuclear family	60 (84.5)	11 (15.5)	1.375 (0.601–3.147)	0.451
	Extended Family	120 (88.2)	16 (11.8)	Ref	
Gravidity	Primigravida	34 (85.0)	6 (15.0)	1.227 (0.46–3.272)	0.683
	Multigravida	146 (87.4)	21 (12.6)	Ref	
GA at the time of abortion	**< 14 weeks**	**128 (84.8)**	**23 (15.2)**	**2.336 (0.77–7.085)**	**0.134**
	14–27 weeks	52 (92.9)	4 (7.1)	Ref	
Type of Abortion	**Induced**	**45 (78.9)**	**12 (21.1)**	**2.4 (1.046–5.508)**	**0.039**
	Spontaneous	135 (90.0)	15 (10.0)	Ref	
History of previous abortion	Yes	52 (85.2)	9 (14.8)	1.231 (0.519–2.916)	0.637
	No	128 (87.7)	18 (12.3)	Ref	
Place where Abortion was performed	**Home**	**90 (84.1)**	**17 (15.9)**	**2.054 (0.843–5.005)**	**0.113**
	**Health center**	**3 (60.0)**	**2 (40.0)**	**7.25 (1.052–49.957)**	**0.044**
	Hospital	87 (91.6)	8 (8.4)	Ref	
Level of service provider	**self**	**64 (82.1)**	**14 (17.9)**	**2.589 (0.939–7.138)**	**0.066**
	**aunt**	**2 (66.7)**	**1 (33.3)**	**5.917 (0.466–75.094)**	**0.17**
	Nurse	43 (87.8)	6 (12.2)	1.651 (0.501–5.445)	0.41
	Doctor	71 (92.2)	6 (7.8)	Ref	
Methods employed	Oral tablets	100 (85.1)	17 (14.9)	0.701 (0.074–6.658)	0.757
	Herbal medications	35 (85.4)	6 (14.6)	0.686 (0.065–7.235)	0.754
	D & C	41 (93.2)	3 (6.8)	0.293 (0.024–3.513)	0.333
	None	4 (80.0)	1(20.0)	Ref	
History of contraceptive use	**No**	**77 (76.2)**	**24 (23.8)**	**10.701 (3.109–36.832)**	**< 0.001**
	yes	103 (97.2)	3 (2.8)	Ref	

Abbreviations: cOR = Crude odds ratio, CI = Confidence interval, D&C = Dilatation and curettages, Ugx = Ugandan shillings

* *p* < 0.2.

**Table 4:** Multivariable analysis of factors associated with septic abortion among women admitted for abortion care at Ugandan satellite teaching hospitals (N = 207).

Variables	Categories	cOR (95% CI)	*p*‐value	aOR (95%CI)	*p*‐value
Residence	**Rural**	**3.52 (1.276–9.709)**	**0.015**	**3.775 (1.056–13.488)**	**0.041** *
	urban	Ref		Ref	
Occupation	Housewife	0.552 (0.1–3.059)	0.496	0.485 (0.075–3.129)	0.447
	Peasant	0.945 (0.243–3.674)	0.935	0.506 (0.109–2.348)	0.384
	Business	0.111 (0.011–1.145)	0.065	0.096 (0.008–1.1)	0.060
	Students	4 (0.788–20.316)	0.095	2.783 (0.42–18.427)	0.288
	Professional	Ref		Ref	
History of contraceptive use	**No**	**10.701 (3.109–36.832)**	**< 0.001**	**6.986 (1.933–25.25)**	**0.003** *
	yes	Ref		Ref	

Abbreviations: aOR = adjusted odds ratio, CI = confidence interval.

* *p* < 0.05.

## 4 Discussion

In the present study, the overall prevalence of septic abortion was 13% (27/207). This finding is consistent with reports from other low‐ and middle‐income countries, such as 13.7% in Malawi [23], 12% in Bangladesh [24], 18.7% in Nigeria [25], and 13.8% in Ethiopia [26]. These comparable rates may reflect shared sociodemographic characteristics, limited access to safe abortion services, restrictive legal environments, and similar cultural and economic contexts. Collectively, these findings underscore the persistent burden of septic abortion as a common complication of unsafe abortion practices across sub‐Saharan Africa and other resource‐constrained settings [27]

Conversely, higher prevalence rates have been reported in certain contexts. For example, 20% of cases have been reported in Ghana [28], and 22.5% have been reported in Iran [7]. These elevated figures may be attributed to variations in healthcare infrastructure, delays in seeking care, restrictive abortion laws, and inadequate access to comprehensive postabortion services.

In contrast, lower prevalence rates have also been documented. For example, 8% reported in Mozambique [29], and 2.4% reported in Tanzania [9]. These findings may illustrate the potential impact of robust reproductive health systems, enhanced contraceptive access, and more enabling legal and policy environments that support safer abortion care. Overall, the variation in reported prevalence across studies highlights the complex interplay between healthcare system capacity, legal context, cultural norms, and women′s access to timely and safe reproductive health services.

In this study, *E. coli* and *S. aureus* were the most frequently isolated pathogens, which is consistent with previous reports from Ethiopia and Nigeria [20, 30]. *E. coli*, a common gastrointestinal commensal, can readily colonize the female genital tract due to its close anatomical proximity, and its endotoxin production is a well‐recognized contributor to septic shock and maternal mortality [13, 20]. Although *Klebsiella spp*. were isolated less frequently than *E. coli* and *S. aureus* in the present study, this finding contrasts with that of Gadisa et al., who reported *Klebsiella spp*. as the predominant isolate [31]. Such differences may reflect regional variations in microbial ecology, healthcare practices, and antimicrobial use patterns. The polymicrobial nature of septic abortion is well documented, with infections arising from endogenous genital tract flora as well as sexually transmitted pathogens [32].

*Escherichia coli* isolates demonstrated complete susceptibility to imipenem and piperacillin–tazobactam, with high susceptibility also observed for chloramphenicol and vancomycin. However, substantial resistance was noted against ciprofloxacin, gentamicin, ampicillin, cefotaxime, and azithromycin. These findings are consistent with those reported by Mwakyoma et al. [33]. The observed resistance to gentamicin in our setting may be influenced by local prescribing practices and widespread empirical use of aminoglycosides [34].

*Klebsiella* spp. isolates exhibited marked resistance to most of the tested antibiotics, particularly ampicillin, cefotaxime, augmentin, gentamicin, azithromycin, and ciprofloxacin. Nevertheless, imipenem and vancomycin retained excellent activity, indicating their continued effectiveness against multidrug‐resistant *Klebsiella* strains. This resistance pattern is in agreement with previous reports [16], although some studies have documented moderate susceptibility to ciprofloxacin, suggesting regional variations in antimicrobial resistance profiles [35].

*Staphylococcus aureus* showed high sensitivity to imipenem, piperacillin–tazobactam, vancomycin, chloramphenicol, and azithromycin, while resistance was mainly observed against augmentin, ampicillin, ciprofloxacin, and cefotaxime. These findings closely mirror those reported by Admas et al. [20], emphasizing the continued effectiveness of glycopeptides and carbapenems against Gram‐positive organisms in this setting.

*Citrobacter spp*. isolates were susceptible to imipenem, gentamicin, piperacillin–tazobactam, and vancomycin but demonstrated resistance to ciprofloxacin, chloramphenicol, ampicillin, cefotaxime, augmentin, and azithromycin. These results correspond with findings by Patience et al. [16]. However, contrasting reports of gentamicin resistance elsewhere may reflect differences in antibiotic usage patterns and levels of empiric therapy [36].

*Enterococcus spp*. showed resistance to cefotaxime, azithromycin, and gentamicin, while maintaining susceptibility to ciprofloxacin, chloramphenicol, imipenem, piperacillin–tazobactam, augmentin, and ampicillin. Although Fouks et al. reported high sensitivity to piperacillin–tazobactam [37], the resistance observed in some settings, particularly to ciprofloxacin, may be attributed to widespread self‐medication and antibiotic misuse [38, 39].

Finally, *Morganella spp*. demonstrated susceptibility to ciprofloxacin, chloramphenicol, imipenem, piperacillin–tazobactam, and augmentin, but resistance to ampicillin, cefotaxime, vancomycin, azithromycin, and gentamicin. These findings are in agreement with those reported by Patience et al. and Liu et al., who highlighted the increasing emergence of beta‐lactam resistance among *Morganella* species, underscoring their growing clinical importance [16, 40].

This study also explored factors associated with septic abortion among postabortion women. Multivariate analysis identified place of residence and previous contraceptive use as significant predictors, highlighting the role of sociodemographic and reproductive health factors in the occurrence of septic abortion.

Women from rural areas had a significantly higher likelihood of experiencing septic abortion (COR = 3.775, 95% CI: 1.056–13.488, *p* = 0.041). This finding is consistent with studies from Zimbabwe, Malawi, and India, which reported higher prevalence rates of septic abortion in rural settings [23, 41, 42]. This association is likely due to limited access to healthcare facilities, leading to delayed medical care [23]. However, some studies did not find a significant link between rural residence and septic abortion [24, 43, 44]

Additionally, women with no history of contraceptive use were seven times more likely to experience septic abortion (COR = 6.986, 95% CI: 1.933–25.25, *p* = 0.003). Similar findings were reported in studies from India, Bangladesh, Nigeria, and Cameroon [16, 45, 46, 47]. This association may be attributed to inadequate knowledge or access to contraceptive methods, which increases the risk of unintended pregnancies and septic abortion [46]. However, some studies did not find an association between the non‐use of contraception and septic abortion [48, 49]

Non‐use of contraception may be linked to unintended pregnancy and therefore, to unsafe induced abortion pathways, which could confound the association between contraceptive non‐use and septic abortion. However, contraceptive non‐use may also reflect pregnancy intention, highlighting the need to interpret this association in the broader context of fertility intentions, access to contraception, and unsafe abortion risk [3].

### 4.1 Study Strengths and Limitations

This is the first documented study to assess the antibiotic susceptibility profiles and determinants of septic abortion among women admitted for abortion care in Ugandan satellite teaching hospitals.

We did not test for Chlamydia trachomatis or anaerobic organisms due to limited laboratory capacity, lack of molecular diagnostics, and absence of anaerobic culture capability. Because these pathogens can contribute to genital tract infection, their exclusion may have underestimated the microbial diversity associated with septic abortion.

## 5 Conclusions and Recommendations

The prevalence observed is comparable to reports from several LMIC settings, though higher than some regional estimates, reflecting ongoing challenges such as limited access to safe abortion, delays in care‐seeking, and suboptimal management of abortion complications. Endocervical cultures were polymicrobial, with *E*. *coli* and *S*. *aureus* being the most common isolates. These pathogens showed high sensitivity to imipenem, piperacillin–tazobactam, chloramphenicol, and vancomycin.

Rural residence and lack of contraceptive use were significantly associated with septic abortion. We recommend strengthening reproductive health education, expanding access to safe abortion services, and improving early care‐seeking. The aforementioned antibiotics, which are readily available in our setting, should be considered for empiric treatment while awaiting culture results. Women from rural areas and those not using contraception warrant targeted preventive and clinical attention.

## Author Contributions

**Guled Bashir Gaboose, Marie Pascaline Sabine Ishimwe**, and **Theoneste Hakizimana:** conceptualization, methodology. **Richard Mulumba** and **Theodore Nteziyaremye:** investigation, data curation. **Theoneste Hakizimana:** formal analysis, interpretation. **Guled Bashir Gaboose, Theodore Nteziyaremye**, and **Marie Pascaline Sabine Ishimwe:** writing – original draft preparation. **Ahmed Kiswezi Kazigo**, **Richard Mulumba**, and **Theoneste Hakizimana:** writing – review and editing. **Theoneste Hakizimana** and **Richard Mulumba:** supervision. All authors have read and approved the final manuscript.

## Funding

The authors received no specific funding for this work.

## Disclosure

All authors have read and approved the final version of the manuscript. Dr. Theoneste Hakizimana had full access to all the data in this study and takes complete responsibility for the integrity of the data and the accuracy of the data analysis.

## Ethics Statement

This research project was approved by the research ethics committee of Kampala International University and the administration of HRRH and FRRH Hospitals under registration number KIU‐REC‐2022‐ 135. The study was registered with the Uganda National Council for Science and Technology (UNCST). All study participants provided written informed consent. All ethical standards were followed according to the Declaration of Helsinki.

## Consent

The authors have nothing to report.

## Conflicts of Interest

The authors declare no conflicts of interest.

## Transparency Statement

The lead author, Theoneste Hakizimana, affirms that this manuscript is an honest, accurate, and transparent account of the study being reported; that no important aspects of the study have been omitted; and that any discrepancies from the study as planned (and, if relevant, registered) have been explained.

## Data Availability

The dataset that was utilized in this study is not publicly available due to ethical considerations. Upon reasonable request, the dataset used can be accessed with the permission of the corresponding author Dr Theoneste Hakizimana (email: theonestehakizimana5@gmail.com).
